# New Coagulant Proteases for Cheesemaking from Leaves and Latex of the Spontaneous Plant *Pergularia tomentosa*: Biochemical Characterization of Coagulants and Sensorial Evaluation of Cheese

**DOI:** 10.3390/foods12132467

**Published:** 2023-06-23

**Authors:** Imene Leulmi, Mohammed Nasreddine Zidoune, Kahina Hafid, Fairouz Djeghim, Hayat Bourekoua, Dariusz Dziki, Renata Różyło

**Affiliations:** 1 Equipe de Transformation et d’Elaboration des Produits Agro-Alimentaires, Laboratoire de Nutrition et de Technologie Alimentaire, Institut de la Nutrition, de l’Alimentation et des Technologies Agro-Alimentaires, Université des Frères Mentouri-Constantine 1, Route de Ain El-Bey, Constantine 25000, Algeria; imane.leulmi@doc.umc.edu.dz (I.L.); zidounem@yahoo.fr (M.N.Z.); fairouze.djeghim@umc.edu.dz (F.D.); bourekoua.hayat@umc.edu.dz (H.B.); 2 Equipe Maquav, Laboratoire de Recherche Biotechnologie et Qualité des Aliments, Institut de la Nutrition, de l’Alimentation et des Technologies Agro-Alimentaires, Université Frères Mentouri-Constantine 1, Route de Ain El-Bey, Constantine 25000, Algeria; kahina.hafid@umc.edu.dz; 3 Department of Thermal Technology and Food Process Engineering, University of Life Sciences in Lublin, 31 Głęboka Street, 20-612 Lublin, Poland; 4 Department of Food Engineering and Machines, University of Life Sciences in Lublin, Głęboka 28, 20-612 Lublin, Poland; renata.rozylo@up.lublin.pl

**Keywords:** *Pergularia tomentosa*, plant proteases, caseinolytic activity, milk-clotting activity, cheese, sensory analysis

## Abstract

The purpose of this study was to evaluate the caseinolytic and milk-clotting activities of aqueous crude extracts from leaves and latex of the *Pergularia tomentosa*, to determine their suitability as a rennet substitute. These extracts were subjected to a series of biochemical tests before being used in the production of cheese. The results showed that the enzymatic latex extract had a higher coagulant activity than the leaf extract. However, under different clotting conditions (pH, temperature, and CaCl_2_ concentration), both coagulants behaved similarly in the coagulation of Berridge substrate. The SDS-PAGE and zymographic analysis revealed identical protein bands with a single active zone in both extracts, corresponding to a molecular weight of 26.98 kDa and 26.03 kDa in the extract of leaf and latex, respectively. Both extracts were stable to different effectors but strongly inhibited by iodoacetamide and Hg, suggesting it to be a cysteine protease. Both extracts were able to hydrolyze casein and generate peptides of 14 kDa, with excessive hydrolysis of the other casein fractions. The physicochemical parameters of cheese made from latex and leaf extract evolved similarly to control cheese. According to the sensory evaluation, cheese made with latex had a mildly bitter flavor but showed a high acceptance rate (>80%).

## 1. Introduction

Cheese is one of the oldest manufactured foods in the human diet. Indeed, the main interest of converting milk into cheese is to preserve the basic components of milk while creating new products with palatable taste, different sensorial characteristics, bioactive compounds, and high nutritional value [1]. Coagulation is the crucial step in the production of all types of cheese; it consists of the transformation of liquid milk into gel following physicochemical modifications occurring on the casein micelles, and it is often the result of the action of proteases of different origins [2].

Rennet, an aspartyl protease that is located in the fourth stomach of unweaned calves, is the oldest coagulant used to enable this transformation. Rennet, however, is unable to satisfy the cheese market for a variety of reasons, including economic, dietary, and religious ones [3]. This prompted the search for alternatives to animal rennet in the form of coagulating enzymes [4]. Since they can be active over a wide range of temperatures and pH, plant proteases have attracted extensive attention from researchers [5]. Coagulant proteases have been extracted and purified from different plant parts belonging to several families [6]. 

Plants belonging to the Asclepiadaceae family are characterized by their latex content. This latex is a mixture of various hydrolytic enzymes, including proteases, which has made it the focus of many researchers in the search for new sources of coagulants [7]. *Pergularia tomentosa* L., also known as “ghalga” in southern Algeria, is one of the plants in this family [8]. It is known for its various medicinal uses among the inhabitants of the region [9]. It is widespread in North Africa, more common in tropical regions and in the Algerian Sahara [10]. This plant is commonly used as a medicinal plant for the treatment of hemorrhoids, bronchial–pulmonary and digestive troubles, and rheumatism [11]. In addition, the presence of latex is one of the important characteristics of this plant. Previous studies carried out with the aqueous extract from *Pergularia tomentosa* indicated the presence of several hydrolytic enzymes such as proteinases, rennin, polyphenol oxidase, tyrosinase, β-amylase, lipase, and L-asparaginase, widespread in the latex compared to the crude extract of the whole plant [12].

However, an evaluation of the milk-clotting and proteolytic activities of this plant’s latex extract as new alternative rennet has not previously been reported. Benyahia et al. [13] conducted the only study on the use of *Pergularia tomentosa* plant leaf extract in the production of fresh cheese. In light of the above, the current study describes for the first time the extraction and characterization of the caseinolytic and coagulant activities of aqueous enzymatic extracts from leaves and latex of the *Pergularia tomentosa*. The effects of various parameters including temperature, pH, CaCl_2_, ions, class-specific protease inhibitors, and kinetic parameters were all tested on caseinolytic and milk coagulation activities. The influence of these extracts on total casein was also studied. These extracts were used to make miniature cheeses, and the physicochemical and sensory properties of the cheese produced were determined.

## 2. Materials and Methods

### 2.1. Reagents and Chemicals

All the reagents and biological substrates used in this work were purchased from SIGMA Chemical Co. (St. Louis, MO, USA) and were of high purity, as buffers were prepared with bi-distilled water. The skimmetod milk powder and the commercial rennet (Rhodia food, Marshall TM, Lyon, France) were a donation from the Safilait food industry (Constantine, Algeria). Rennet is characterized by a Soxhlet strength of 1/100,000 and contains 520 mg of chymosin in 1 g of powder. The fresh cow’s milk was obtained from a private farm in Algeria. Salt was purchased from the local market.

### 2.2. Plant Material and Latex Collection

The plant material used in this study (*Pergularia tomentosa* L.), was harvested from the In Aminas region of Illizi City, Algeria at a geolocation of 28°02′18″ N 9°33′54″ E. The plant grows spontaneously along the roadsides and in areas near water.

The fresh latex from the *Pergularia tomentosa* plant was obtained byincising the fruits and leaves from their stems, collecting the cast latex in clean containers and storing at −20 °C until use.

### 2.3. Preparation of Enzymatic Crude Extracts

Fresh leaves were carefully cleaned and washed with water, air-dried, and kept in the dark for 1 week with constant turning to avoid microbial development and alterationof the leaves. Triplicate samples of 25 g of the dried leaves were ground and macerated in 100 mL of sodium acetate buffer (0.1 M–pH 5.5) for 6 h at 4 °C under stirring. After that, extract was filtered through gauze, the filtrate was centrifuged for 10 min at 6000× *g* at 4 °C, and the supernatant was dialyzed overnight against an acetate buffer pH 5.5 at 0.1 M (10 kDa cutoff). The extract obtained, corresponding to the crude extract of leaves, was stored at−20 °C.

The crude latex extract was prepared as described by Freitas et al. [14] with some modifications. Briefly, the recovered latex was mixed with distilled water at a ratio of 1:1 (*v*/*v*), and then centrifuged at 10,000× *g* for 10 min at 4 °C to remove rubber and other insoluble impurities.The recuperated supernatant was dialyzed overnight against distilled water (10 kDa cutoff).The recovered dialyzed crude latex extract was stored at −20 °C.

### 2.4. Protein Quantification

The protein content in both extracts was determined according to the Bradford assay [15], using a standard solution of bovine serum albumin (BSA) at 2% (*w*/*v*).

### 2.5. Caseinolytic Activity Measurement

Casein hydrolysis was performed, as described by Hafid et al. [16] with slight modifications using bovine casein (1% *w*/*v*) as substrate in a 50 mM phosphate buffer (pH 7.5). Briefly, 0.1 mL of each crude enzymatic extract diluted in 0.7 mL of buffer was added to 0.8 mL of casein solution. After 10 min of reaction at 37 °C, casein hydrolysis was stopped by adding 1.6 mL of 10% (*w*/*v*) trichloroacetic acid (TCA) solution. The mixture was left to stand at room temperature for 15 min, and then centrifuged at 6000× *g* for 10 min. The absorbance of the supernatant was measured at 280 nm. One unit (U) of caseinolytic activity was defined as the amount of enzyme capable to generate an increase in absorbance by 0.001 min^−1^ at 280 nm according to the following formula:

(1) $$\mathrm{Caseinolytic}\;\mathrm{activity}\;\left(\frac{U}{\mathrm{mL}}\right)=\frac{\Delta A280\;\mathrm{nm}\;\times \;\mathrm{DF}}{t}0.001,$$

where ΔA280 nm = A280 nm (test) − A280 nm (control without enzyme), DFis the dilution factor, tis the reaction time (10 min), and 0.001 is a factor to explain the change in extinction.

### 2.6. Electrophoretic Protein Profiles

The electrophoretic profile of the proteins contained in crude extracts was carried out using tricine SDS-PAGE, using 12% separating and 4% stacking gels. Briefly, the samples were treated at a ratio of 1:1 (*v*/*v*) with denaturing buffer containing 100 mM Tris-HCl (pH 6.8), 1% (*w*/*v*) SDS, 0.75% (*w*/*v*) DTT, 0.02% (*w*/*v*) Coomassie Blue Brilliant R250, and 20% (*w*/*v*) glycerol, and then warmed at 75 °C for 10 min. Ten micrograms of protein samples were loaded on to the gel and separation was then subjected for 4 h at 140 V. When separation was achieved, the gel was stained with Coomassie Blue R250. Protein bands were visualized after destaining the gel. The molecular weights of protein bands were estimated using the UN-SCAN-IT gel 6.5 analysis software (Silk Scientific, Orem, UT, USA).

### 2.7. Zymography

To detect the protein bands with proteolytic activity in enzymatic extracts, zymography was performed as described by Gagaoua et al. [17], using the same SDS-PAGE conditions. The protein bands in the gel were renatured by incubation in Triton X-100 at 2.5% (*v*/*v*) for 30 min, and then washed twice with deionized water. The gel was incubated in 50 mL of 2% casein in 50 mM phosphate buffer (pH 7.0) for 2 h, and then the gel was stained and destained. The appearance of a clear and white zone in the gel reveals a band with proteolytic activity.

### 2.8. Effect of Temperature, Thermal Stability, and pH on Caseinolytic Activity

The temperature effect on caseinolytic activity was carried out as described above at various temperatures from 40 °C to 90 °C with 10 °C intervals [16]. The relative activities as percentages were expressed as the ratio of enzymatic activity obtained at a certain temperature to the maximum activity obtained at the given temperature range.

The thermal stability of enzymatic extracts was determined by measuring the residual activity of the enzyme maintained at the same temperatures for 1 h. After desired incubation periods, enzyme aliquots were withdrawn and assayed at optimal assay conditions to determine the residual enzyme activity.

The effect of pH on proteolytic activity was performed under the same standard assay procedure by varying the pH range from 3.5 to 9, using the following buffers (50 mM): citrate buffer (pH 3.5–6.0), phosphate buffer (pH 6.5–7.5), and Tris-HCl (pH 8–9) [16].

### 2.9. Effect of Metal Ions and Protease Inhibitors on Caseinolytic Activity

To study the influence of various metal ions (KCl, NaCl, CuSO_4_, ZnSO_4_, MnSO_4_, HgSO_4_, Fe (SO_4_)_3_, AlCl_3_, CaCl_2_, and MgCl_2_) and specific protease inhibitors (β-mercaptoethanol (BME), ethylenediaminetetraacetic acid (EDTA), iodoacetamide (IAA), and phenyl-methylsulfonylfloride (PMSF)) on the caseinolytic activity of enzymaticextracts, the different solutions of metal ions at 2 mM or protease inhibitors (5 mM) were preincubated with the enzymatic extract for 30 min at 37 °C, and then the reaction was performed as previously mentioned, followed by measuring the residual activity using the casein assay as described above. The enzyme activity assayed in the absence of metal ions or inhibitor was considered as control and defined as 100%. The inhibition percentage was expressed as the residual activity compared to the measured activity without effectors.

### 2.10. Kinetic Parameters

Kinetic parameters K_m_ and V_max_ of proteases were determined by measuring the proteolytic activity at progressive concentrations of casein from 0 to 50 mg/mL as a specific substrate. Then the activity was estimated under the same conditions as previously described. The Michaelis–Menten and Lineweaver–Burk curves (reciprocal) were acquired using the Sigma Plot (Ver. 12.0, Systat Software Inc., San Jose, CA, USA) to calculate K_m_ and V_max_ [16].

### 2.11. Determination of Milk-Clotting Activity and Optimal Coagulation Conditions

The milk-clotting activity (MCA) was determined according to Arima and Iwasaki [18], with modification. Briefly, 10 mL of the standard Berridge substrate (12% *w*/*v* skim milk powder dissolved in 0.01 M CaCl_2_ solution, pH 6.4) was preincubated for 5 min at 37 °C, and 1 mL of enzymatic extract was added. Test tubes were periodically rotated by hand until appearance of visible discrete particles to the naked eye. MCA was defined as the amount of enzyme contained in 1mL that coagulated 10 mL of the substrate within 40 min (2400 s) at 37 °C, expressed as

(2) $$\mathrm{MCA}\;\left(U/\mathrm{mL}\right)=\left(\frac{2400}{\mathrm{clotting}\;\mathrm{time}\;\left(s\right)}\right)\times \mathrm{dilution}\;\mathrm{factor}.$$

The ratio between milk-clotting activity and proteolytic activity (milk-clotting index, MCI) was also calculated according to the following formula:

(3) $$\mathrm{MCI}=\frac{\mathrm{MCA}}{\mathrm{PA}}.$$

To determine the optimal coagulation conditions by enzymatic extracts of *Pergularia tomentosa*, the effects of temperature, pH, and CaCl_2_ concentration on MCA were investigated. The effect of temperature was determined following the standard assay procedure in the temperature range from 40 °C to 90 °C with an increment of 10 °C, and the MCA was measured at a constant pH of 6.4 and CaCl_2_ concentration at 0.01 M.

The effect of pH on the MCA of enzymatic extracts was determined at 37 °C by varying the milk pH from 5.5 to 8.5.

For the influence of CaCl_2_ concentration on MCA, the concentration of CaCl_2_ ions in milk varied from 0.008 M to 0.06 M and the flocculation time was measured using the standard assay procedure.

### 2.12. SDS-PAGE Analysis of Casein Hydrolysis by Enzymatic Extracts

The protein profile of casein fractions generated under the action of proteases from latex and leaf extracts of *P. tomentosa* was observed by 15% SDS-PAGE according to Laemmli [19] as described by Freitas et al. [20]. Caseins were dissolved in 0.1 M phosphate buffer (pH 6.5) containing 0.1% (*w*/*v*) sodium azide to a final concentration of 1% (*w*/*v*). The reaction was started by mixing 50 μL of enzyme and 450 μL of substrate at 40 °C. Aliquots (50 µL) were taken at different time intervals (0, 1, 5, 10, 15, 30, 60, 90, and 120 min). The reaction was stopped by mixing each aliquot with Tris-HCl (pH 6.8) SDS-PAGE sample buffer containing 0.1% SDS, 5% β-mercaptoethanol, 10% glycerol, and 0.01% bromophenol blue (150 µL), followed by heating at 90 °C for 5 min. Microbial chymosin was used as a control (50 μL 0.1 mg/mL in distilled water).

### 2.13. Cheesemaking

Three cheese samples were prepared at a laboratory scale using the extracts of latex, filtrate of *Pergularia tomentosa* leaves and commercial chymosin in cow’s milk. Neither CaCl_2_ nor starter culture was added for cheesemaking. For each manufacture, 2.5 L of cow’s milk was used.

The preparation of curds by the leaf extract was performed according to the manufacturing diagram of the wagashi cheese produced with the leaf extract of *Calotropis procera* as described by Rayanatou et al. [21].

**Figure 1:**
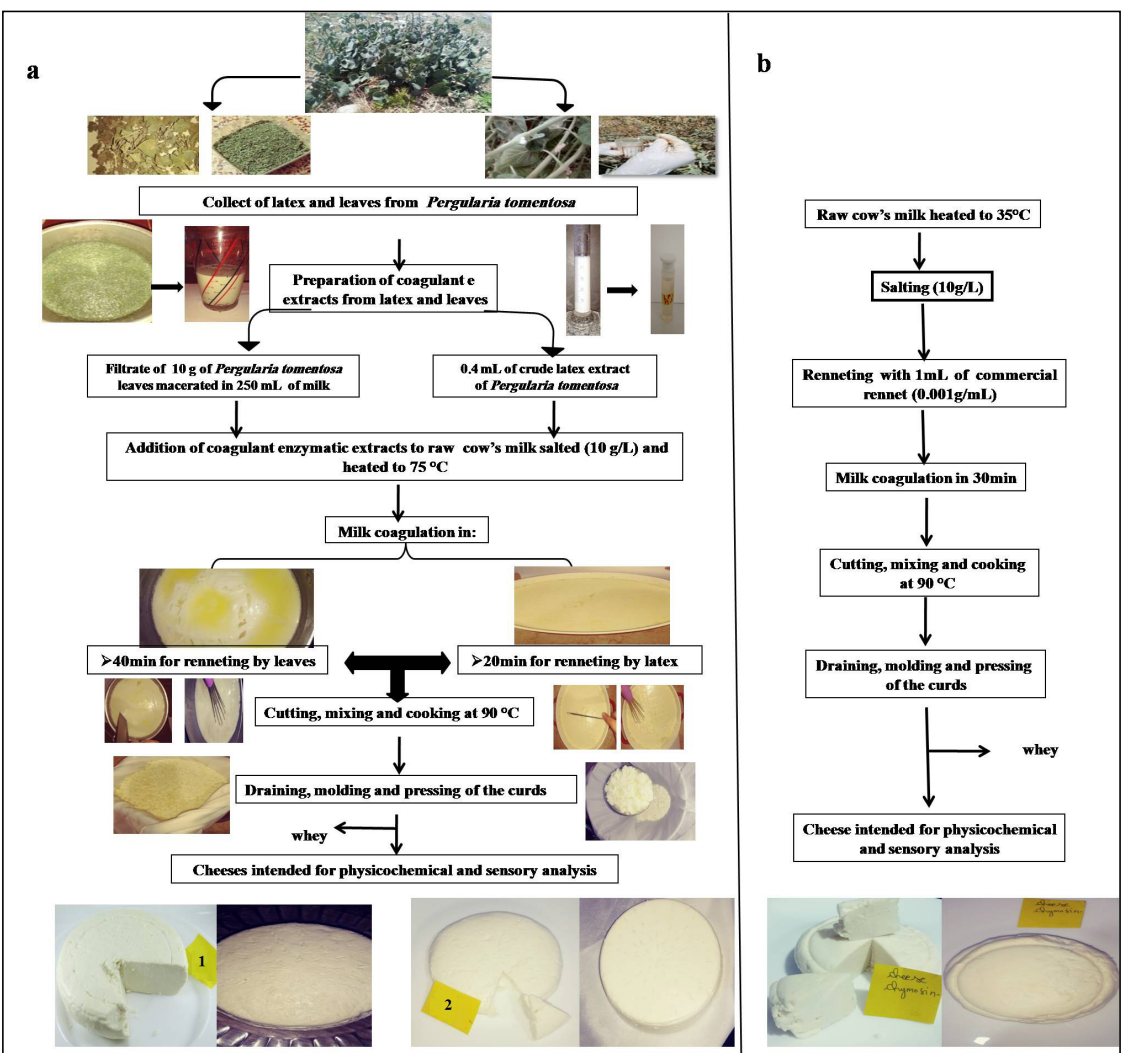
Cheesemaking diagram with different coagulant extracts: (**a**) with leaves and latex extracts (1: cheese made with leaf extract; 2: cheese made with latex extract); (**b**) with commercial rennet.

The coagulant was prepared by soaking 10 g of the dry leaf powder in 250 mL of the milk. After 1 h of incubation at 25 °C, the mixture was filtered through a white cloth to remove leaf debris, and then added to milk that was heated at 75 °C. On the other hand, the cheese curd made with latex was prepared by adding of 0.4 mL of crude extract to 2.5 L of cow milk heated at 75 °C. For chymosin, the same manufacturing procedure was used, except that the milk was heated at 35 °C. Coagulation was completed in 20 min using latex extract, and 40 min using filtrate of leaf extract. After coagulation, the curds were left to firm up for 20 min, and then manually cut into cubes and stirred for 20 min; next, the temperature was increased again to 90 °C for 10 min to cook the curds and promote the syneresis. After constant stirring for 10 min, the curd was separated from the whey, placed in plastic molds, and then pressed for 10 h. The diagram of cheesemaking is presented in Figure 1.

#### 2.13.1. Physicochemical Evaluation

The manufactured cheeses were analyzed for total solids, moisture, and lactic acid content according to the standard methods of AFNOR [22]. The pH values of the cheeses were determined using a digital pH meter. Fat content was measured following the method of Gerber [23]. Cheeses yield was also calculated; the fresh yield of the cheese was expressed according to Vacca et al.’s [24] formula:

(4) $$\mathrm{Fresh}\;\mathrm{curd}\;\mathrm{yield}\;\left(\%\right)=\left(\frac{\mathrm{mass}\;\mathrm{of}\;\mathrm{fresh}\;\mathrm{crud}}{\mathrm{mass}\;\mathrm{of}\;\mathrm{milk}}\right)\times 100.$$

#### 2.13.2. Sensory Evaluation

Descriptive sensory analysis was carried out to appreciate the organoleptic properties and estimate the degree of acceptability of produced cheeses by consumers. A consumer-based rating test was performed by 10 trained testers (28–46 years old, five females and five males). Attributes related to color, appearance, texture, smell, taste, flavor, overall assessment, and acceptance index were evaluated. The sensory analysis report that was distributed to the panel included a list of definitions for all of the attributes. Each attribute was evaluated using a predefined scale (0: absence of perception, 9: very intense perception) [25]. For each panelist, three cubes of cheese samples were presented.

### 2.14. Statistical Analysis

The mean and standard deviation (SD) for three replicates were calculated in this study unless otherwise stated. Means were compared and evaluated using analysis of variance followed by Fisher’s least significant difference post hoc test. A statistical difference at *p* < 0.05 was considered significant. Data were statistically analyzed using XLSTAT (Addinsoft, Paris, France).

## 3. Results and Discussion

### 3.1. Extraction and Enzymatic Activity Characteristics of Pergularia tomentosa Extracts

The latex from *Pergularia tomentosa* used in this work contains approximately 12% (*v*/*v*) of the gums. The resulting supernatant had a pH of 5.85 ± 0.1; it was whitish in color and viscous in texture, representing 88% (*v*/*v*) of the total latex volume, and it contained a protein content of 31.44 mg/mL (Table 1). This value was similar to that obtained by Shivaprasad et al. [26] in the latex of *Pergularia extensa* with a value of 30 mg/mL.

The proteolytic activity of the new proteases endowed with coagulant activity is of paramount importance to estimate the possibility of their use in the manufacture of cheese as a rennet substitute. The latex extract showed strong proteolytic activity of 161.66 ± 3.77 U/min with a specific activity estimated at 5.14 U/mg. This activity is higher than that reported by Shivaprasad et al. [26] in the latex of *Pergularia extensa*, which was 135 U/min, and in the latex of *Calotropis gigantea*, estimated at 86.45 U/min [27]. This finding is in agreement with results found by Shivaprasad et al. [7] in the latex enzyme fractions extracted from different plants belonging to the Asclepiadaceae family, *Asclepias curassavica, Calotropis gigantea, Pergularia extensa*, and *Cynanchum puciflorum,* where they found a specific proteolytic activity of 4, 3, 4.5, and 5 U/mg/min respectively.

On the other hand, leaf extract revealed a protein content of 5.70 ± 0.24 mg/mL, a yield of 22.8 mg of protein per 1 g of the dry leaf (2.28% m/m), and a proteolytic activity of 24.66 ± 0.44 U/min.There wasno statistically significant difference between the specific proteolytic activities of the leaf extract and that of the latex. This could be explained by the fact that, in addition to the proteases, the amount of protein recorded in the latex extract contains other proteins or enzymes.

### 3.2. Electrophoresis Profile and Zymography

**Figure 2:**
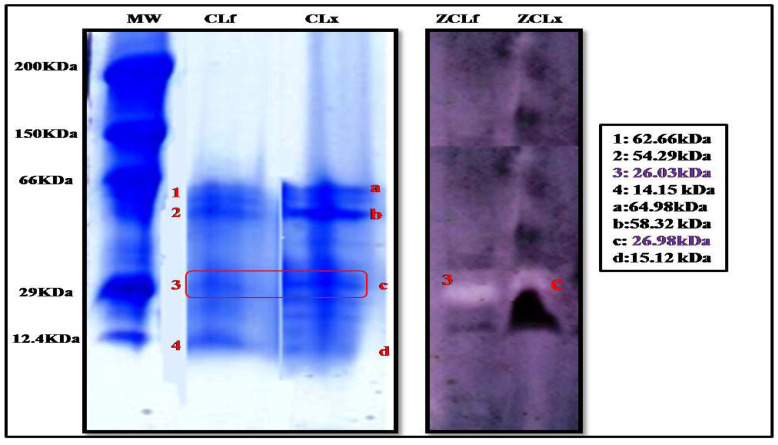
Tricine SDS-PAGE of *Pergularia tomentosa* plant crude extracts (10% separating polyacrylamide gel) (MW:molecular weight standards; CLf: crude leaf extract, and CLx: crude latex extract; ZCLf: zymogram of crude leaf extract; ZCLx: zymogram of crude latex extract) (3 and C: protein bands with proteolytic activity).

The electrophoretic profile by tricine SDS-PAGE electrophoresis (Figure 2) of the latex and leaf extracts showed a clear similarity in the content of the extracts, where we noticed the presence of four protein bands in both extracts. However, they were more intense in the latex extract than in the leaf, leading us to conclude that proteins were more concentrated in the latex than in the leaves.

These protein bands were also subjected to zymographic analysis, using casein as a substrate. The zymogram gel (Figure 2) showed one resolved band that exhibited a proteolytic activity corresponding to a molecular mass of 26.98 kDa and 26.03 kDa in the extract of latex and leaf, respectively, suggesting the monomeric nature of the enzyme. These findings are supported by those reported by Shivaprasad et al. [26] who reported a molecular weight of 26 kDa for the protease extracted from *Pergularia extensa* latex.

### 3.3. Effect of Temperature, Thermal Stability, and pH on Proteolytic Activity

**Figure 3:**
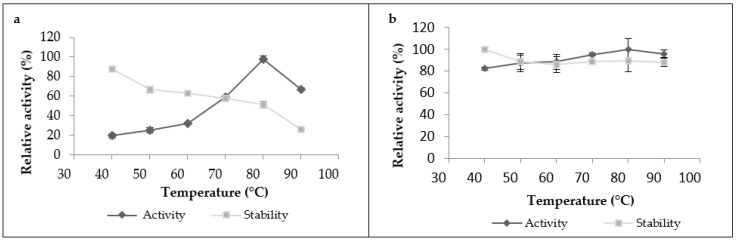
Temperature’s effect on proteolytic activity and stability of *Pergularia tomentosa* extracts ((**a**): leaf extract, (**b**): latex extract).

The proteolytic activity of leaf extract significantly increased with heating until it reached its maximum at 80 °C (Figure 3a), and then began to decline after heating at 90 °C. The crude extract was also stable under a wide range of temperatures; it kept more than 60% of its initial activity in the temperature range from 40 to 70 °C when it was incubated for 1 h, and it retained 90% of its initial activity at 40 °C. However, it lost 75% of its activity when incubated at 90 °C for 1 h. As for the latex extract, it was very active and stable over a broad temperature range and reached a maximum activity at 80 °C. It was active even at 90 °C without losing its initial activity after 1 h of incubation, showing great thermostability (Figure 3b).These findings showed that proteases from this plant exhibited high optimal temperatures and thermostability compared to the other proteases plant. Referring to existing studies on plant proteases, we found that most of them have optimal temperatures ranging between 40 and 60 °C [16]. Nevertheless, a similar optimum temperature at 70 °C was shown in partially purified milk-clotting enzyme from *Solanum dubium* Fresen seeds reported by Ahmed et al. [28]. This high stability could also be explained by the fact that this plant grows indesert areas, where it can withstand high temperatures without losing effectiveness. Another important factor that can be added to explain this great thermostability is the natural structure of plant proteases and the degree of glycosidic bonds present in the structure proteases, which have strong thermal stability. According to Duarate et al. (2006), glycosylation is a secondary expression which gives the plant better stability of its proteins, resistance to degradation and, thus, adaptationtoits external environment. On the basis of this factor, the observed differences in thermal stability observed between latex extract and leaf extract could be explained by the degree of glycosidic bonds, which can be in a greater proportion in the latex protease than in the leaf protease.

**Figure 4:**
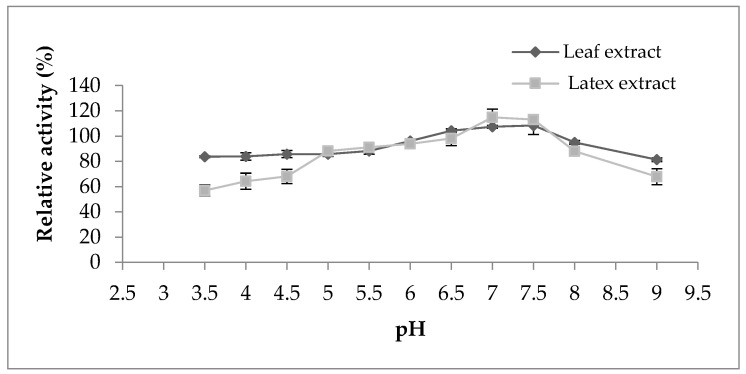
Effect of pH on proteolytic activity of *Pergularia tomentosa* extracts.

Regarding the pH effect on the proteolytic activity (Figure 4), both extracts represented the same behavior displaying maximum activity in the pH range from 6 to 7.5; outside of this range, the activity began to decline. However, the activity of both extracts was not strongly affected by the change in pH, which indicates their stability in a large pH range. According to the literature bibliography, the majority of proteases belonging to the milkweed family have an optimal pH between 5 and 8, as observed in papain [16], procerain B [29], and philibertain g I [30] where they marked an optimum pH of 7, 8, and 7, respectively.

### 3.4. Effect of Metal Ions and Protease Inhibitors on Caseinolytic Activity

**Figure 5:**
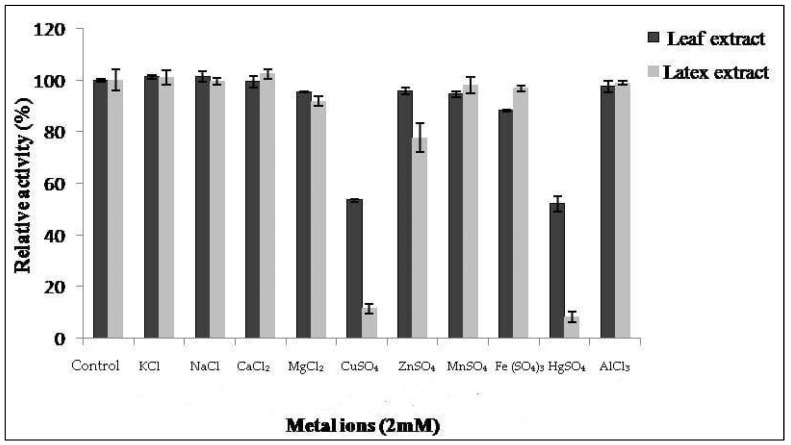
Effect of ions on activity of *Pergularia tomentosa* extracts.

The proteolytic activity of both extracts from latex and leaves was monitored in the presence of various metal ions at 2 mM of concentration (Figure 5). Proteolytic activity of latex was affected by 86% and 90% in the presence of CuSO_4_ and HgSO_4_ ions, respectively. This inhibition was also observed by Golden and Smith-Marshall [31] on theproteolytic activity of the bromelain-like enzyme from Noniinhibited by mercury chloride (0.1 mM). Asimilar inhibition profile was found by Badgujar and Mahajan [32] in the latex of *Euphorbia nivulia*. The inhibition of proteases by mercury ion Hg^2+^ indicates the presence of amino acids containing a thiol(–SH)function at or near the active site [33]. This leads us to suppose that *Pergularia tomentosa* extracts contain a thiol group, probablymaking them cysteine proteases.

The results show thatCaCl_2_, NaCl, AlCl_3_, MnSO_4_, and KCl metals had no significant inhibitory effect on the proteolytic activity of our extracts. Similar results were reported in [20,34], where they showed that NaCl and CaCl_2_ do not have an inhibitory effect. On the other hand, MgCl_2_ and ZnSO_4_ exhibited a slight inhibitory effect on the activity of latex and FeSO_4_ ion on the activity of leaf extract.

**Figure 6:**
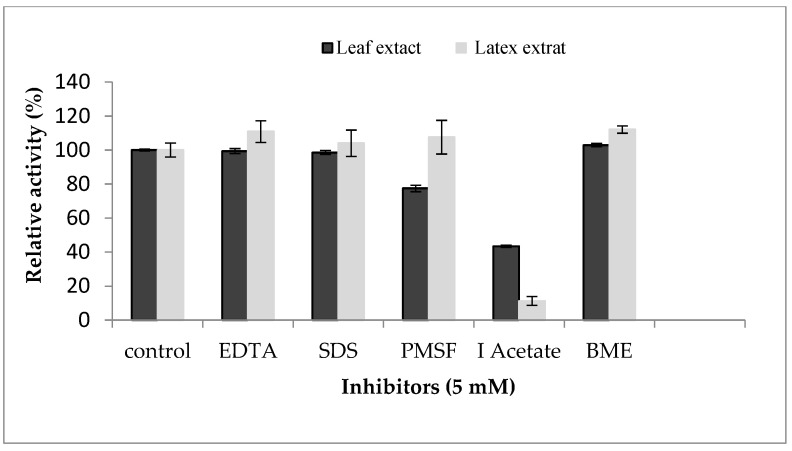
Effect of protease inhibitors on proteolytic activity of *Pergularia tomentosa* extracts.

In order to determine the nature of proteases contained in the enzymatic extracts of the *P. tomentosa* plant, an inhibition study was realized (Figure 6). The resultsreveal that the leaf extract was inhibited by both IAA and PMSF, losing 57% and 23% of its enzymatic activity, respectively, indicating the coexistence of cysteine and serine proteases. Similar results were reported by [27] for leaf, flower, and stem extracts of *Calotropis gigantea*. On the other hand, latex extract was inhibited strongly (90%) by IAA at 5 mM. No inhibition was observed in the presence of PMSF or EDTA, specific inhibitors of serine proteases and metalloproteases, respectively. These results exclude the possibility that the proteaseof *Pergularia tomentosa* latex is a metal or serine protease. Inhibition by IAA suggested the presence of a unique type of cysteine proteases in latex. As we saw above, proteolytic activity was inhibited in presence of mercuric sulfate at 2 mM. This inhibition profile confirms that proteases from *P. tomentosa* latex could be a member of the cysteine protease class. A similar inhibition profile was also established in various latexes extracted from Asclepiadaceae plants that were inhibited over 90% by IAA suggesting the presence of a unique type of cysteine proteases [7]. In addition to that, several other latexes contain cysteine proteases inhibited by IAA such as papain [16], ervatamin A, ervatamin B, funastrain c II, morrenain bI, and philibertain gI [35].

These findings also support what we found previously in electrophoresis and zymogramresults, as most of the previous studies proved that cysteine proteases have molecular weights ranging between 21 and 30 kDa [36].

### 3.5. Kinetic Parameters

**Figure 7:**
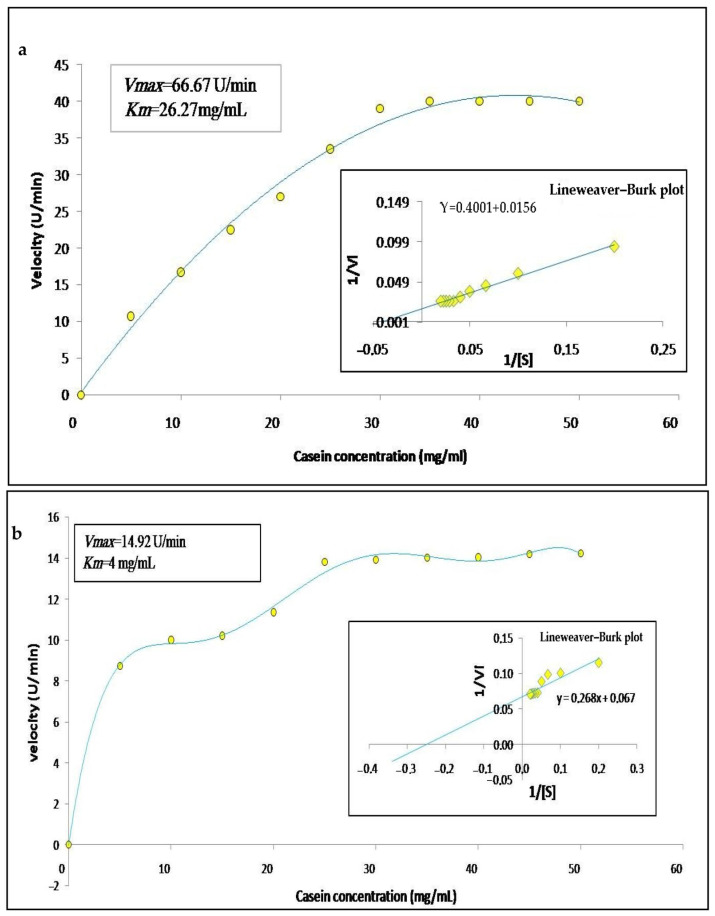
Graphical representations of Michaelis–Menten and Lineweaver–Burk of *Pergularia tomentosa* extracts ((**a**): latex extract, (**b**): leaf extract).

The enzymatic activity of the crude extracts was calculated according to the concentration of substrate in order to determine the substrate specificity, as well as their kinetic parameters; thus, we varied the concentration of casein from 0 to 50 mg/mL. The graphic representation in Figure 7a,b, shows that the enzymes follow a simple Michaelian allure, indicating that they are monomeric enzymes, as already observed in the zymogram. The Line weaver–Burk plot (Figure 7a,b) allowed finding a Michaelis constant K_m_ of 26.27 mg/mL and 4 mg/mL and maximum velocity V_max_ of 66.67 U/min and 14.29 U/min for latex and leaf extracts, respectively. These results are similar to those described by Hafid et al. [16] who obtained hyperbolic kinetics for papain a cysteine protease extracted from *Carica papaya*. Low values of K_m_ indicate a high affinity of an enzyme for a substrate. Moreover, high values of V_max_ indicate catalytic efficiency.

### 3.6. Milk-Clotting Activity

The milk-clotting activity of crude extracts from latex and leaves of *P. tomentosa* was investigated. The results indicate that enzymatic extract from latex exhibited an MCA of 1246.45 U/mL against 97.17 U/mL givenby leaf extract (Table 1). These results are similar to those reported by [27]. The specific milk-clotting activity (SMCA) of latex (38.18) was twice that of the leaf extract (17.04). This parameter was calculated to evaluate the ability of any new coagulant to be used in cheesemaking. Thus, the milk-clotting index (MCI) as the ratio of milk-clotting activity to proteolytic activity (MCA/PA) was 3.96 ± 0.3 and 7.45 ± 0.68 for the extracts of leaves and latex, respectively. This ratio is important because it is used as a quality characteristic of coagulants, whereby a good coagulant should reveal high SMCA and low specific proteolytic activity (SPA) [20]. In addition, the MCA/PA is directly related to cheese yield, one of the most important economical characteristics in the cheesemaking process. The MCI of both extracts was considered tolerable for cheesemaking by previous studies. Overall, the MCI of most plant coagulants is reported to be in the range of 0.68 to 9.58 [37]. Results found in the present study are consistent with other research demonstrating that plant proteases frequently have a lower MCI than bovine chymosin [38]. Anusha et al. [27] showed that the MCI from different plant parts of *C. gigantea* ranged from 0.622 to 5.21. Silva et al. [39] reported that CpCP_3_ exhibited an MCI of around 8.4. This is because the majority of milk-clotting proteases from plants possess a higher proteolytic activity, which leads to weakness in the cheese yield and alterations in the overall appearance of curds.

**Figure 8:**
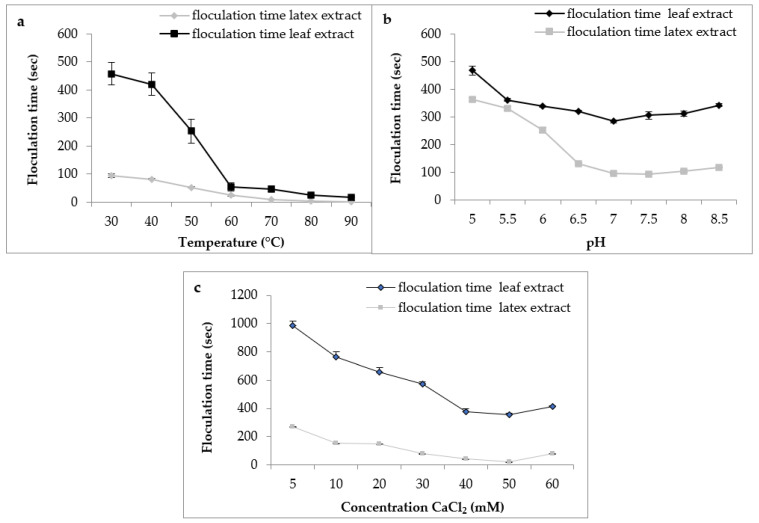
Effect of milk temperature (**a**), pH (**b**), and CaCl_2_ concentration (**c**) on flocculation time of *Pergularia tomentosa* enzymatic extracts.

Regarding the effect of temperature, the results (Figure 8a) indicated a decrease in the flocculation time of Berridge substrate by both extracts used in this study with increasing temperature. We note that the shape of the curve was similar for both extracts with an optimum temperature marked in the interval of 75 °C and 90 °C, from where there was instantaneous flocculation.

These findings are consistent with those reported by various authors, who stated that plant proteases are exceptionally thermostable and have an optimal temperature of activity that is greater than that of animal proteases [40]. Protease from *C. procera* leaves was more active at 65 °C for clotting cow milk [41]. The milk-clotting protease from *Cynara scolymus* exhibited maximum activity at 70 °C for clotting cow milk [42].

For the effect of pH on flocculation time, the results (Figure 8b) indicate that both extracts had the same behavior according to the pH of milk. Both extracts showed maximum activity in a range of pH 6.5 to 8 with an optimal activity at pH 7, suggesting that these plant extracts are more active at neutral pH, and confirming what we found previously on proteolytic activity. Similar results were reported by Hafid et al. [16] on papain from *C. papaya* that had an interesting milk clotting activity at pH 6.5.

Calcium chloride CaCl_2_ is usually used in the cheese industry to increase the firmness of rennet curds and reduce coagulation time [43]. Figure 8c depicts the effect of CaCl_2_ concentration on the flocculation time of milk. The curve shape was similar in both extracts. Therefore, the gradual increase in the concentration of CaCl_2_ led to a decrease in the flocculation time and, therefore, an increase in the milk-clotting activity. In our working conditions, the CaCl_2_ concentration of 50 mM was considered the optimal concentration. Then, at higher concentrations of 60 mM, the flocculation time of crude extracts was increased. Other clotting plant enzymes yielded similar results [20,44]. The decrease observed in the coagulation activity at higher CaCl_2_ concentrations can supposedly be explained by the change in ionic strength or saturation of the negatively charged residues of casein micelles caused by the increase of Ca^2+^ in the medium [45]. Although it is necessaryto promote milk coagulation, several reports have shown that high levels of CaCl_2_ can reduce milk pH, impair the aggregation of the micelles, and possibly decrease proteolytic activity [46].

### 3.7. Effect of Pergularia tomentosa Protease Extracts on Caseins Hydrolysis

**Figure 9:**
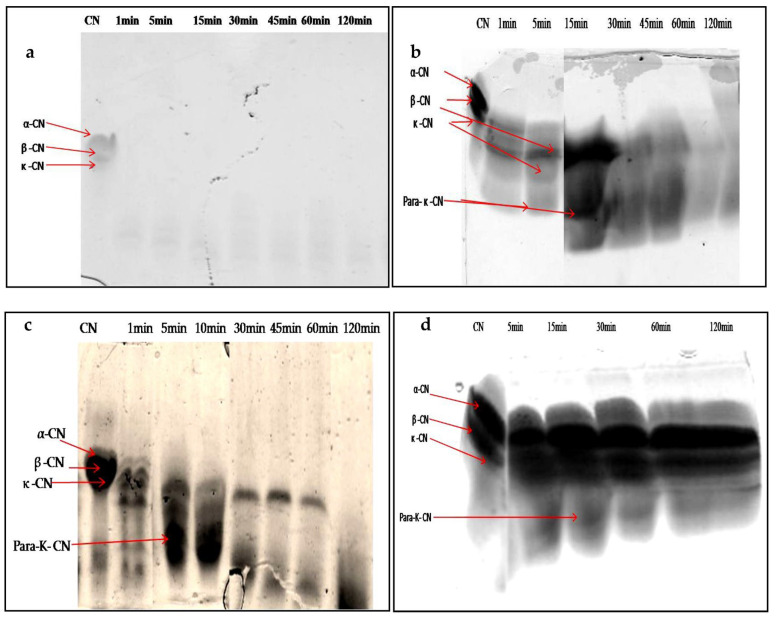
SDS-PAGE (12%) pattern of hydrolysis of the total bovine casein by latex extract of *P. tomentosa* ((**a**): undiluted extract; (**b**): diluted extract to 1/200 *v*/*v*; (**c**): leaf extract; (**d**): commercial rennet (line 1: native casein, lines 2–7: times of incubation 1 min, 5 min, 10 min, 30 min, 45 min, 60 min, and 120 min)).

When characterizing any new rennet substitute for use in cheesemaking, it is crucial to assess the degradation patterns of the caseins, due to their impact on the yield, flavor, and texture of the final cheese [47]. The SDS-PAGE electrophoresis technique was used to follow the degradation profiles of total casein at different times of incubation. Figure 9a–d show the digestion profile of total caseins by the crude extracts of latex (undiluted and diluted extract at 1/200 *v*/*v*) and leaf, as well as by the microbial chymosin. The hydrolysis profile of total caseins by microbial chymosin (Figure 9d) showed that only κ-casein was hydrolyzed and revealed a band of approximately 14 kDa, corresponding the *para*-κ-casein after 30 min of incubation. This band resulted from the hydrolysis of κ-casein at the Phe105–Met106 peptide bond, which represents the first enzymatic step of milk coagulation. Moreover, the bonds in αs-casein and β-casein seemed to be unaffected after 120 min of incubation, showing a specific action of chymosin toward κ-casein. On the other hand, the electrophoretic profile of casein hydrolysis by undiluted latex extract showed the immediate and extensive hydrolysis of total casein. It degraded all casein fragments in 1 min of enzymatic reaction, and no bands were detected (Figure 9a). The gradual degradation of casein was observed when latex extract was diluted at 1/200 (*v*/*v*) (Figure 9b). Electrophoresis profiles (Figure 9b,c) showed that the digestion of αs-casein and κ-casein by both extracts was achieved after 5 min of reaction, with the appearance of other bands with lower molecular weights less than 20 kDa released after 15 min, including a peptide band of approximately 14 kDa, likely the *para*-κ-casein, suggesting that both enzymatic extracts cleaved κ-casein similarly to microbialchymosin rennet.

Whereas β-casein showed a lowest mobility and remained intact for up to 30 and 60 min for latex and leaf extracts, respectively, the band intensity disappeared after 60 min of incubation, and then casein bands disappeared completely after 120 min of incubation with both crude extracts. The hydrophobic nature of β-casein probably made it more resistant to hydrolysis than αs-casein.

These results indicate that extracts prepared from *P. tomentosa* plant exhibited unspecific hydrolysis on total casein. Moreover, it was found that the hydrolysis including para- κ-caseinwas degraded after a long incubation; in contrast, the microbial rennet was specific and the band of *para*-κ-casein remained intact. It should also be noted that the intensity of degradation of αs- and β-caseins depended on the concentration of enzyme used, as well as the time of hydrolysis.

Thishydrolysis showed that the proteases contained in the extracts of latex and leaves of *P. tomentosa* plant gave new bands which certainly corresponded to the fragments of peptides resulting from the digestion of casein, which confirmed the nonspecific action of these proteases toward casein like all plant proteases, as well as explained the great proteolytic activity obtained. Furthermore, this result could encourage the research on peptides with biological activity.

Similar studies have been carried out on the coagulant enzymes of *Solanum dubium* [28], *Albizia julibrissin* [48], and *Cynara cardunculus* [49]; these authors showed that the enzymes of these plants are responsible for the primary hydrolysis of total caseins into peptides of low molecular weight responsible for the bitterness.

These results indicate that proteases from *P. tomentosa* plant can be used as an alternative to rennet but it is necessary to control the concentration of enzymes and the coagulation time to prevent excessive hydrolysis of casein, which is the main obstacle found in the use of plant proteases for cheesemaking; these steps may affect the taste and texture of cheeses, resulting in an excessively acidic or bitter taste and weak yield.

### 3.8. Characteristics of Produced Cheese

As shown in Table 2, there was no statistically significant difference (*p* > 0.05) in the pH values of the three types of cheese, where the pH of the curd tended to be neutral. This could be explained by the fact that no lactic bacteria were added during the manufacturing process; in addition, it could be due to the initial pH of milk used. A similar result was reported by Rayanatou et al. [21] who estimated a pH of 6.52 in curd produced with *C. procera* leaves.

Regarding acidity and fat, the use of enzymatic extracts from the latex and leaves of *P. tomentosa* gave values that are similar to those found in the control curd. This, moreover, was confirmed by several authors who reported that the acidity and fat content of cheeses depended only on the nature and initial composition of the milk [50,51].

However, a significant difference was noted in the rate of total solids between the cheeses made with latex extract and those made with leaf extract and microbial chymosin. According to Alais [52], total dry extract varies by cheese type; it is influenced by the initial composition of the milk, the type of coagulation, and the type of draining.

Moisture content is an important parameter because it plays a major role in the protein arrangement of curd and, thus, affects cheese hardness [53]. The moisture content found for leaf cheese (53.81%) is lower than that recorded by Rayanatou et al. [21] in the cheese made using leaves extract of *C. procera*, with a moisture content of 69%. Omotosho et al. [54] and Silva et al. [55] reported a moisture content of 50% and 54% for cheese made with latex extract of *C. procera* in contrast to the 60.66% recorded in this work for cheese made with latex extract. Cheese made with latex was softer than cheese made with leaf extract and microbial rennet.

The cheese yield is of great interest in the cheese industry, because it globally reflects the quantitative distribution of the constituents of the milk during draining, thus allowing to judge whether the production has been carried out under good conditions [56]. Cheese made with latex yielded less than cheese made with microbial chymosin. Higher moisture (60.66%) confirmed this. The use of a mixture of crude enzymatic extracts, which can have several disadvantages for cheesemaking, such as extensive hydrolysis of caseins, especially α- and β-casein, causing low yield and bitter taste, could explain the lower yield [3].

### 3.9. Sensory Characteristics of Produced Cheese

Making a cheese that is acceptable to the consumer must consider not only the ability of protease to curdle the milk but also quality and sensory accessibility criteria. The coagulating agent, according to Walstra et al. [43], is involved in determining the sensory characteristics of cheeses. Cheese made with plant extracts was compared to cheese made with commercial rennet. A descriptive test was carried out for this purpose.

**Figure 10:**
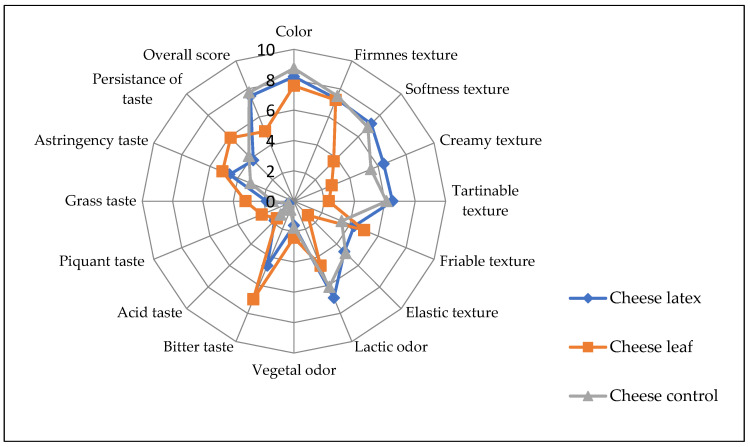
Sensory profile of cheeses made with different coagulants from *Pergularia tomentosa* latex extract, dried leaf extract, and microbial chymosin. 0 to 9: perception scale (0: absence of perception, 9: very intense perception).

In terms of color and smell, the ANOVA showed that there was no significant difference (*p* > 0.05) among the three kinds of cheese produced with different coagulating agents. In general, the consumers did not report a statistically significant difference between the cheese obtained by the latex extract and that of the control cheese for most of the sensory attributes except for the bitter taste, where the latex cheese had a bitterness score of 4.6 (Figure 10). In addition, the astringency taste is more pronounced in leaves and latex cheeses. Cheese made with leaf extract, on the other hand, was bitterer than cheese made with latex extract. This can be attributed to the fact that plant leaf extract also contains, in addition to proteases, other substances such as polyphenols, tannins, and alkaloids, which have a bitter taste and a green color, leading to an undesirable side-effect on the final organoleptic characteristics of the cheese and reducing its acceptability by the consumer [57]. To mitigate this disadvantage, the leaf extract should be purified before use. The bitter taste can also be reduced by diluting the plant extract, which results in a longer coagulation time and less de-concentration of the other bitter composites. Because of the other sensorial characteristics, cheese made with leaf extract is generally acceptable.

The coagulant agent seems to have affected the tasters’ overall rating. For the cheeses made with latex extract, microbial rennet, and leaf extract, the calculated acceptance index (AI) for the overall acceptance of each cheese was 80.55, 86.1, and 51.88, respectively. A product is considered to be good if its acceptance index is greater than 70%, according to [58]. Cheese made with latex extract did not show a significant difference with control cheese.

## 4. Conclusions

Enzymatic extracts of leaves and latex of the *Pergularia tomentosa* plant were investigated and characterized for the first time in this work. Overall, the results obtained in this study seem promising, showing that extracts from latex and leaves of the *P. tomentosa* plant contain cysteineproteases capable of replacing rennet.The cheeses obtained by using latex extract showed a great similarity to the cheese control with the presence of a slightly bitter taste, but it gave a good acceptance index. The popularization of this plant and its extracts can contribute to socioeconomic development and fill the deficit in protein in the desert area. Further studies on the enzymatic extracts of the *P. tomentosa* are required to master its proteolytic activity and, thus, determine the type of cheese corresponding to its enzymatic activity, as well as its use in other food industries, e.g., as meat-tenderizing agents.

## Figures and Tables

**Table 1:** Physicochemical characteristics of crude enzymatic extracts from latex and leaves of *Pergularia tomentosa* plant.

Parameter	Leaf Extract	Latex Extract
Yield (%)	75.66 ± 0.88 ^a^	88.00 ± 2.00 ^b^
pH	6.00 ± 0.10 ^a^	5.85 ± 0.10 ^a^
Protein content (mg/mL)	5.70 ± 0.24 ^a^	31.44 ± 1.76 ^b^
Proteolytic activity (U/min)	24.66 ± 0.44 ^a^	161.66 ± 3.77 ^b^
Specific proteolytic activity (U/mg)	4.32 ± 0.25 ^a^	5.14 ± 0.31 ^a^
MCA (U/mL)	97.92 ± 4.90 ^a^	1246.45 ± 69.01 ^b^
Specific milk-clotting activity (U/mg)	17.26 ± 1.54 ^a^	39.89 ± 4.27 ^b^
MCI (MCA/PA)	3.96 ± 0.16 ^a^	7.70 ± 0.35 ^a^

^a, b^ A significant difference in the same line at 0.05 level.

**Table 2:** Biochemical characteristics of cheeses produced with different extracts.

Parameter	Curd Latex	Curd Leaf	Curd Chymosin
pH	6.41 ± 0.02 ^a^	6.44 ± 0.02 ^a^	6.73 ± 0.01 ^a^
Acidity% (g/100 g)	0.33 ± 0.90 ^a^	0.35 ± 0.20 ^a^	0.32 ± 0.018 ^a^
Total solids (*w*/*w*)	39.34 ± 1.14 ^a^	46.19 ± 0.31 ^b^	47.28 ± 0.39 ^b^
Moisture (g/100 g)	60.66 ± 1.14 ^a^	53.81 ± 0.31 ^b^	52.72 ± 0.39 ^b^
Fat %(*w*/*w*)	21.50 ± 0.30 ^a^	22.00 ± 0.20 ^a^	21.40 ± 0.10 ^a^
Cheese mass for 1 L of milk (g)	200.45	238.20	227
Cheese yield %	19.51	23.22	22.14

Different letters show statistical significance (*p* < 0.05).

## Data Availability

The data used to support the findings of this study can be made available by the corresponding author upon request.

## References

[1] López-Expósito I., Miralles B., Amigo L., Hernández-Ledesma B. (2017). Health effects of cheese components with a focus on bioactive peptides. Fermented Foods in Health and Disease Prevention.

[2] Meng F., Chen R., Zhu X., Lu Y., Nie T., Lu F., Lu Z. (2018). Newly Effective Milk-Clotting Enzyme from *Bacillus subtilis* and Its Application in Cheese Making. J. Agric. Food Chem..

[3] Shah M.A., Mir S.A., Paray M.A. (2014). Plant proteases as milk-clotting enzymes in cheesemaking: A review. Dairy Sci. Technol..

[4] Jacob M., Jaros D., Rohm H. (2011). Recent advances in milk clotting enzymes. Int. J. Dairy Technol..

[5] Uhlig H. (1998). Industrial Enzymes and Their Applications.

[6] Mazorra-Manzano M.A., Moreno-Hernández J.M., Ramírez-Suarez J.C. (2018). Milk-clotting plant proteases for cheesemaking. Biotechnological Applications of Plant Proteolytic Enzymes.

[7] Shivaprasad H., Riyaz M., Venkatesh Kumar R., Dharmappa K., Tarannum S., Siddesha J., Rajesh R., Vishwanath B. (2009). Cysteine proteases from the Asclepiadaceae plants latex exhibited thrombin and plasmin like activities. J. Thromb. Thrombolysis.

[8] Chehma A. (2006). Catalogue des Plantes Spontanées du Sahara Septentrional Algérien.

[9] Maroyi A. (2016). Treatment of diarrhoea using traditional medicines: Contemporary research in South Africa and Zimbabwe. Afr. J. Tradit. Complement. Altern. Med..

[10] Cherif R., Kemassi A., Boual Z., Bouziane N., Benbrahim F.B.F., Hadjseyd A., Gharib T., Hadj-Khelil A.O.E., Sakeur M.L., Hadj M.D.O.E. (2016). Activités biologiques des extraits aqueux de *Pergularia tomentosa* L. (Asclepiadaceae). Rev. Laser Eng..

[11] Goodman S.M., Hobbs J.J. (1988). The Ethnobotany of the Egyptian Eastern Desert: A comparison of common plant usage between two culturally distinct Bedouin groups. J. Ethnopharmacol..

[12] Lahmar I., Yotova L. (2016). Investigation of Different Enzyme Activities from *Pergularia tomentosa* L. and *Ecballium elaterium* L.. J. Chem. Technol. Metall..

[13] Benyahia F., Zitoun O.A., Meghzili B., Foufou E., Zidoune M. (2021). Use of *Pergularia tomentosa* Plant Enzymatic Coagulant System in Fresh Cheese-Making. Food Nutr. Sci..

[14] Freitas C.D.T., Oliveira J.S., Miranda M.R.A., Macedo N.M.R., Sales M.P., Villas-Boas L.A., Ramos M.V. (2007). Enzymatic activities and protein profile of latex from *Calotropis procera*. Plant Physiol. Biochem..

[15] Bradford M.M. (1976). A rapid and sensitive method for the quantitation of microgram quantities of protein utilizing the principle of protein-dye binding. Anal. Biochem..

[16] Hafid K., John J., Sayah T.M., Domínguez R., Becila S., Lamri M., Dib A.L., Lorenzo J.M., Gagaoua M. (2020). One-step recovery of latex papain from *Carica papaya* using three phase partitioning and its use as milk-clotting and meat-tenderizing agent. Int. J. Biol. Macromol..

[17] Gagaoua M., Hoggas N., Hafid K. (2015). Three phase partitioning of zingibain, a milk-clotting enzyme from *Zingiber officinale* Roscoe rhizomes. Int. J. Biol. Macromol..

[18] Arima K., Yu J., Iwasaki S. (1970). Milk-clotting enzyme from *Mucor pusillus* var. Lindt. Methods in Enzymology.

[19] Laemmli U.K. (1970). Cleavage of Structural Proteins during the Assembly of the Head of Bacteriophage T4. Nature.

[20] Freitas C.D.T., Leite H.B., Oliveira J.P.B., Amaral J.L., Egito A.S., Vairo-Cavalli S., Lobo M.D.P., Monteiro-Moreira A.C.O., Ramos M.V. (2016). Insights into milk-clotting activity of latex peptidases from *Calotropis procera* and *Cryptostegia grandiflora*. Food Res. Int..

[21] Rayanatou I.A., Mahamadou E.G., Garric G., Harel-Oger M., Leduc A., Jardin J., Briard-Bion V., Cauty C., Adakal H., Grongnet J.F. (2017). Physico-chemical characterization of dairy gel obtained by a proteolytic extract from Calotropis procera—A comparison with chymosin. Food Chem..

[22] Amariglio S. (1986). Contrôle de la Qualité des Produits Laitiers, Analyses Physiques et Chimiques.

[23] James C. (1995). Determination of the fat content of dairy products by the Gerber Method. Analytical Chemistry of Food.

[24] Vacca G.M., Stocco G., Dettori M.L., Pira E., Bittante G., Pazzola M. (2018). Milk yield, quality, and coagulation properties of 6 breeds of goats: Environmental and individual variability. J. Dairy Sci..

[25] Peryam D.R., Pilgrim F.J. (1957). Hedonic scale method of measuring food preferences. Food Technol..

[26] Shivaprasad H.V., Rajaiah R., Frey B.M., Frey F.J., Vishwanath B.S. (2010). ’Pergularain e I’—A plant cysteine protease with thrombin-like activity from *Pergularia extensa* latex. Thromb. Res..

[27] Anusha R., Singh M.K., Bindhu O. (2014). Characterisation of potential milk coagulants from *Calotropis gigantea* plant parts and their hydrolytic pattern of bovine casein. Eur. Food Res. Technol..

[28] Ahmed I.A.M., Morishima I., Babiker E.E., Mori N. (2009). Characterisation of partially purified milk-clotting enzyme from *Solanum dubium* Fresen seeds. Food Chem..

[29] Singh A.N., Shukla A.K., Jagannadham M., Dubey V.K. (2010). Purification of a novel cysteine protease, procerain B, from *Calotropis procera* with distinct characteristics compared to procerain. Process Biochem..

[30] Sequeiros C., Torres M., Trejo S., Esteves J., Natalucci C., López L. (2005). Philibertain g I, the most basic cysteine endopeptidase purified from the latex of *Philibertia gilliesii* Hook. et Arn. (Apocynaceae). Protein J..

[31] Golden K., Smith-Marshall J. (2012). Characterization of bromelain from *Morinda citrifolia* (Noni). J. Sci. Res..

[32] Badgujar S.B., Mahajan R.T. (2014). Nivulian-II a new milk clotting cysteine protease of Euphorbia nivulia latex. Int. J. Biol. Macromol..

[33] De Farias V.A., da Rocha Lima A.D., Costa A.S., de Freitas C.D.T., da Silva Araújo I.M., dos Santos Garruti D., de Figueiredo E.A.T., de Oliveira H.D. (2020). Noni (*Morinda citrifolia* L.) fruit as a new source of milk-clotting cysteine proteases. Food Res. Int..

[34] Silva A.C.D., Nascimento T.C.E.d.S., Silva S.A.d., Herculano P.N., Moreira K.A. (2013). Potential of quixaba (*Sideroxylon obtusifolium*) latex as a milk-clotting agent. Food Sci. Technol..

[35] Mahajan R.T., Badgujar S.B. (2010). Biological aspects of proteolytic enzymes: A review. J. Pharm. Res..

[36] Grzonka Z., Kasprzykowski F., Wiczk W. (2007). Cysteine proteases. Industrial Enzymes: Structure, Function and Applications.

[37] Brutti C.B., Pardo M.F., Caffini N.O., Natalucci C.L. (2012). *Onopordum acanthium* L. (Asteraceae) flowers as coagulating agent for cheesemaking. LWT Food Sci. Technol..

[38] Mazorra-Manzano M.A., Perea-Gutiérrez T.C., Lugo-Sánchez M.E., Ramirez-Suarez J.C., Torres-Llanez M.J., González-Córdova A.F., Vallejo-Cordoba B. (2013). Comparison of the milk-clotting properties of three plant extracts. Food Chem..

[39] Silva M.Z.R., Oliveira J.P.B., Ramos M.V., Farias D.F., de Sá C.A., Ribeiro J.A.C., Silva A.F.B., de Sousa J.S., Zambelli R.A., da Silva A.C. (2020). Biotechnological potential of a cysteine protease (CpCP3) from Calotropis procera latex for cheesemaking. Food Chem..

[40] Claverie-MartÌn F., Vega-Hernàndez M.C., Polaina J., MacCabe A.P. (2007). Aspartic Proteases Used in Cheese Making. Industrial Enzymes: Structure, Function and Applications.

[41] Aworh O.C., Nakai S. (1986). Extraction of Milk Clotting Enzyme from Sodom Apple (*Calotropis procera*). J. Food Sci..

[42] Sidrach L., García-Cánovas F., Tudela J., Rodríguez-López J.N. (2005). Purification of cynarases from artichoke (*Cynara scolymus* L.): Enzymatic properties of cynarase A. Phytochemistry.

[43] Walstra P., Wouters J.T.M., Geurts T.J. (2005). Dairy Science and Technology.

[44] Pontual E.V., Carvalho B.E., Bezerra R.S., Coelho L.C., Napoleão T.H., Paiva P.M. (2012). Caseinolytic and milk-clotting activities from Moringa oleifera flowers. Food Chem..

[45] Kumari M., Sharma A., Jagannadham M.V. (2012). Religiosin B, a milk-clotting serine protease from Ficus religiosa. Food Chem..

[46] Wolfschoon-Pombo A.F. (1997). Influence of calcium chloride addition to milk on the cheese yield. Int. Dairy J..

[47] Fox P.F., McSweeney P.L.H., Fox P.F., McSweeney P.L.H., Cogan T.M., Guinee T.P. (2004). Cheese: An Overview. Cheese: Chemistry, Physics and Microbiology.

[48] Otani H., Matsumori M., Hosono A. (1991). Purification and some properties of a milk clotting protease from the young seeds of *Albizia julibrissin*. Animal Sci. Technol..

[49] Sousa M.J., Malcata F.X. (2002). Advances in the role of a plant coagulant (*Cynara cardunculus*) in vitro and during ripening of cheeses from several milk species. Lait.

[50] Aquilanti L., Babini V., Santarelli S., Osimani A., Petruzzelli A., Clementi F. (2011). Bacterial dynamics in a raw cow’s milk Caciotta cheese manufactured with aqueous extract of *Cynara cardunculus* dried flowers. Lett. Appl. Microbiol..

[51] Zikiou A., Zidoune M.N. (2019). Enzymatic extract from flowers of Algerian spontaneous *Cynara cardunculus*: Milk-clotting properties and use in the manufacture of a Camembert-type cheese. Int. J. Dairy Technol..

[52] Alais C. (1984). Science du Lait: Principes des Techniques Laitières.

[53] De Moraes G.M.D., dos Santos K.M.O., de Barcelos S.C., Lopes S.A., do Egito A.S. (2018). Potentially probiotic goat cheese produced with autochthonous adjunct culture of *Lactobacillus mucosae*: Microbiological, physicochemical and sensory attributes. Lwt.

[54] Omotosho O., Oboh G., Iweala E. (2011). Comparative effects of local coagulants on the nutritive value, in vitro multienzyme protein digestibility and sensory properties of Wara cheese. Int. J. Dairy Sci..

[55] Bittante G., Cipolat-Gotet C., Cecchinato A. (2013). Genetic parameters of different measures of cheese yield and milk nutrient recovery from an individual model cheese-manufacturing process. J. Dairy Sci..

[56] Khan A.A., Naqvi T., Naqvi M. (2012). Identification of phytosaponins as novel biodynamic agents: An updated overview. Asian J. Exp. Biol. Sci..

[57] Dutcosky S.D. (2007). Análise sensorial de alimentos. Análise Sensorial de Alimentos.

[58] Duarate P., Figueiredo R., Pereira S., Pissarra J. (2006). Structural characterization of the stigma-style complex of *Cynaracardunculus* (Asteraceae) and immunolocalization of cardosin A and B during floral development. Botany.

